# The Diagnostic and Prognostic Value of the 12-Lead ECG in Arrhythmogenic Left Ventricular Cardiomyopathy

**DOI:** 10.1016/j.jacadv.2025.101766

**Published:** 2025-05-12

**Authors:** Leonardo Calò, Cinzia Crescenzi, Andrea Di Marco, Francesca Fanisio, Fabiana Romeo, Alessio Gargaro, Annamaria Martino, Chiara Cappelletto, Marco Merlo, Mattia Targetti, Elisabetta Toso, Federica Toto, Maria Beatrice Musumeci, Giacomo Tini, Michele Ciabatti, Matteo Stefanini, Stefano Canestrelli, Elisa Fedele, Chiara Lanzillo, Armando Fusco, Federica Carla Sangiuolo, Cinzia Radesich, Maria Perotto, Maurizio Pieroni, Ruggiero Mango, Alessio Gasperetti, Camillo Autore, Michela Casella, Antonio Dello Russo, Davide Stolfo, Mikael Laredo, Estelle Gandjbakhch, Maddalena Graziosi, Elena Biagini, Costantina Catalano, Ludovica Barile, Fabrizio Drago, Marianna Cicenia, Anwar Baban, Gemma Pelargonio, Maria Lucia Narducci, Federica Re, Giovanni Peretto, Elena Paiotti, Carles Diez Lopez, Iacopo Olivotto, Fiorenzo Gaita, Gianfranco Sinagra, Giuseppe Novelli

**Affiliations:** a Division of Cardiology, Policlinico Casilino, Rome, Italy; b Department of Motor, Human and Health Sciences, “Foro Italico” University of Rome, Rome, Italy; c Arrhythmia Unit, Cardiology Department, Bellvitge University Hospital, Barcelona, Spain; d Clinical Research Unit, Biotronik Italia S.P.A, Cologno Monzese MI, Italy; e Cardiothoracovascular Department, Azienda Sanitaria Universitaria Giuliano Isontina (ASUGI), University of Trieste, Trieste, Italy; f Cardiomyopathy Unit, Careggi University Hospital, Florence, Italy; g Division of Cardiology, Department of Medical Sciences, AOU Città della Salute e della Scienza Hospital, University of Turin, Turin, Italy; h Cardiology Unit, Department of Clinical and Molecular Medicine, Sapienza University of Rome - Sant'Andrea University Hospital, Rome, Italy; i Cardiovascular Department, San Donato Hospital, Arezzo, Italy; j Division of Radiology, Policlinico Casilino, Rome, Italy; k Department of Biomedicine and Prevention, Tor Vergata University, Rome, Italy; l Cardiology Unit, Department of Emergency and Critical Care, Policlinico Tor Vergata, Rome, Italy; m Johns Hopkins University, Baltimore, Maryland, USA; n Department of Cardiology and Respiratory Sciences, San Raffaele Cassino (FR), Cassino, Italy; o Cardiology and Arrhythmology Clinic, University Cardiology Hospital Ospedali Riuniti, Ancona, Italy; p Division of Cardiology, Department of Medicine, Karolinska Institutet, Stockholm, Sweden; q Cardiology Institute, Pitié-Salpetriere, AP-HP, Sorbonne Université, Paris, France; r Cardiology Unit, St. Orsola Hospital, IRCCS Azienda Ospedaliero-Universitaria di Bologna, European Reference Network for Rare, Low Prevalence, and Complex Diseases of the Heart (ERN GUARD-Heart), Bologna, Italy; s Pediatric Cardiology and Cardiac Arrhythmias Complex Unit, Bambino Gesù Children's Hospital, IRCCS, Rome, Italy; t Cardiology Institute, Catholic University of the Sacred Heart, Rome, Italy; u Cardiology Division, Cardiac Arrhythmia Center and Cardiomyopathies Unit, St. Camillo-Forlanini Hospital, Rome, Italy; v Department of Cardiac Electrophysiology and Arrhythmology, IRCCS San Raffaele Hospital, Vita-Salute San Raffaele University, Milan, Italy; w Institut de Investigació Biomèdica de Bellvitge (IDIBELL) Barcelona, Spain-Centro de Investigación Biomédica en Red (CIBER-CV), Madrid, Spain; x Cardiology Unit, Meyer University Children Hospital IRCCS, University of Florence, Florence, Italy

**Keywords:** arrhythmias, arrhythmogenic cardiomyopathy, cardiomyopathy, desmoplakin, desmosome, ECG, magnetic resonance imaging, prognosis, sudden cardiac death

## Abstract

**Background:**

Electrocardiographic findings in arrhythmogenic left ventricular cardiomyopathy (ALVC) have been limited to small studies.

**Objectives:**

The authors aimed to analyze the electrocardiogram (ECG) characteristics of ALVC, to correlate ECG with cardiac magnetic resonance and genetic data, and to evaluate its prognostic value.

**Methods:**

We reviewed data of 125 consecutive patients with ALVC (81.5% *desmoplakin* pathogenic/likely pathogenic variants). The composite endpoint of major arrhythmic events (MAEs) included sudden cardiac death, aborted sudden cardiac death, and appropriate implantable cardioverter-defibrillator shock. Predictors of MAE were evaluated with logistic regression.

**Results:**

ALVC showed distinct ECG signs, including left posterior fascicular block (LPFB) (13.6%), pathological Q waves (26.4%), R/S ratio in V_1_ ≥0.5 (26.4%), and SV1 + RV6 ≤12 mm and RI + RII ≤8 mm (44%). Fifteen (12%) patients had a normal ECG. MAE occurred in 35 patients (28%). In multivariable analysis, LPFB (OR: 4.7; 95% CI: 1.2-18.3), syncope (OR: 84.95; 95% CI: 14-496), transmural late gadolinium enhancement (OR: 9.95; 95% CI: 2.3-36), and right ventricular ejection fraction (OR: 0.92; 95% CI: 0.87-0.97) were the independent predictors of MAE. The model including these 4 variables achieved a remarkable predictive capability (area under the curve: 0.9). In the primary prevention scenario, with Cox regression, LPFB (HR: 3.98; 95% CI: 1.3-12.0), syncope (HR: 19.13; 95% CI: 5.8-63.0), and transmural late gadolinium enhancement (HR: 10.57; 95% CI: 2.9-38.0) were independent predictors of MAE.

**Conclusions:**

In ALVC, ECG is a valuable diagnostic tool and may have a relevant prognostic role, since LFPB is a strong and independent predictor of MAE.

In recent years, some studies have analyzed the phenotype and the genetic features of arrhythmogenic left ventricular cardiomyopathy (ALVC), even though this cardiomyopathy is yet to be completely described.^1^, ^2^, ^3^, ^4^, ^5^, ^6^, ^7^, ^8^, ^9^, ^10^, ^11^, ^12^, ^13^, ^14^, ^15^, ^16^, ^17^, ^18^, ^19^, ^20^ Few investigations have analyzed the electrocardiogram (ECG) findings in ALVC.^3^, ^4^, ^5^, ^6^, ^7^^,^^19^^,^^20^ However, limited data have been published regarding the relationship between genotype, ECG, and late gadolinium enhancement (LGE) location, pattern, or distribution at cardiac magnetic resonance (CMR). A prevalent subepicardial LGE distribution in inferior and lateral left ventricle (LV) walls has been reported.^2^, ^3^, ^4^, ^5^, ^6^, ^7^, ^8^^,^^18^^,^^19^ In patients affected by nonischemic dilated cardiomyopathy (DCM), a typical subepicardial, ring-like LGE pattern was observed, particularly in those with *desmoplakin (DSP)* and filamin-C genotypes.^8^ Regarding the outcome, syncope and right ventricular ejection fraction (RVEF) have been reported as relevant risk factors for major arrhythmic event (MAE) in arrhythmogenic cardiomyopathy.^13^, ^14^, ^15^, ^16^ The prognostic role of ring-like LGE, which has been described as a hallmark of carriers of DSP variants,^8^ has not been previously evaluated in a specific cohort of ALVC. Furthermore, no information is available about the potential prognostic role of ECG in ALVC.

Recently, we described new ECG signs in ALVC, such as the presence of left posterior fascicular block (LPFB), pathological Q-waves in inferior and/or lateral leads, prominent R-wave in V_1_ with a R/S ratio ≥0.5, and a sum of the R-wave ≤8 mm in I to II and S-wave in V_1_ and the R-wave in V_6_ ≤12 mm.^19^

The aims of this study were the following: 1) to confirm in a larger population our prior observations about ECG findings in ALVC; 2) to evaluate the correlation between ECG abnormalities and CMR and genetic data; and 3) to explore the prognostic value of this comprehensive ECG evaluation.

## Methods

### Study population

A retrospective data analysis of patients with ALVC, consecutively referred to 14 European cardiomyopathy clinics from May 1, 2015, to March 31, 2022, was performed. We collected information on family, medical and pharmacological history, ECG, transthoracic echocardiography, Holter-ECG, exercise ECG, CMR, genetic test, autopsy, endomyocardial biopsy, and implantable cardioverter-defibrillator (ICD) reports. Patients without interpretable ECG, with paced rhythm, inadequate CMR data, or without accurate follow-up data were excluded.

The diagnosis of ALVC, characterized by predominant involvement of LV with little or no abnormalities in the right ventricle (RV), was established based on the most recent criteria.^11^ Specifically, the criteria for a definite diagnosis of ALVC included the following:

1. Presence of LGE in the LV, manifesting as a stria (or band) pattern affecting ≥1 segment.
2. Positive genetic testing for pathogenic (class V)/likely pathogenic (class IV) variants in genes responsible for desmosomal proteins associated with ALVC.^13^
3. Confirmation of ALVC diagnosis through endomyocardial biopsy (sample obtained from the LV in one of the areas presenting LGE at CMR) or at autopsy,^19^^,^^21^ for those cases in which genetic screening did not identify any pathogenic mutation, since the current prevalence of pathogenic variants (PVs) found in ALVC probands is approximately 50% to 60%.^12^

The study was approved by the Institutional Review Board (Cardiopatie ARITMOgene [CARITMO] study). All patients gave written informed consent.

### ECG assessment

The ECG tracing recorded (25 mm/s, 1 mV/cm) at the patient's inclusion in the study was used for the analysis. All ECG tracings were manually analyzed by 3 independent cardiologists (L.C., C.C., F.R.) blinded to outcomes of patients and to the CMR data; discrepancies were resolved by consensus. Conduction disturbances and the measurement of QRS complex and PR interval duration followed guidelines.^22^ LPFB was defined by the presence of all the following: 1) frontal plane axis 100° to 180°; 2) rS pattern in leads I and aVL; 3) qR pattern in leads III and aVF; 4) QRS duration <110 ms; and 5) absence of a QS pattern in I and aVL.^23^

The QRS complex components were measured (millimeters) in all leads, and R/S ratio was measured in each lead. The ECG was analyzed for the presence of pathological Q-waves (≥40 ms, or ≥3 mm, or qR ratio ≥0.25), fragmented QRS (fQRS),^24^ and low QRS voltages (LQRSVs), defined as <0.5 mV in limb leads and <1 mV in precordial leads, including both negative and positive components.^25^ A LQRSV in the limb leads was defined when each lead was <0.5 mV. When present both in limb and precordial leads, LQRSV was defined as global. These depolarization ECG parameters were considered abnormal if present in ≥2 contiguous leads except aVR. Since fibrosis in ALVC could involve the LV lateral wall, Tzou^26^ (V_1_ R-wave and V_6_ S-wave ≥0.15 mV) and Bayés de Luna criteria^27^ (R/S ≥0.5 and R >3 mm) were analyzed.

Ventricular repolarization was analyzed in accordance with the American Heart Association/American College of Cardiology/Heart Rhythm Society statement^28^ by: 1) corrected QT interval in lead II (Bazett method); 2) T-wave inversion (TWI) ≥0.1 mV in depth in ≥2 contiguous leads in the absence of complete left bundle branch block or right bundle branch block; and 3) ST-segment depression. Based on data of our previous study,^19^ we considered employing as new diagnostic criteria for ALVC, the sum of the R-wave in I and II ≤8 mm, and the sum of the S-wave in V_1_ and the R-wave in V_6_ ≤12 mm.

When available, we analyzed ECGs recorded during follow-up to assess any changes over time.

### Cardiac magnetic resonance imaging

All studies were acquired within 1 month after enrollment on 1.5-T machines (vendors: General Electric, Philips, Siemens). Examinations included standard cine imaging with steady-state free precession, CMR and LGE analysis was performed as recently described.^19^ LGE pattern was considered as ring-like if there were at least 3 contiguous segments with subepicardial/midmyocardial LGE in the same short-axis slice.^8^ On the basis of the location and pattern of LGE, patients were divided in ring-like and no ring-like pattern.

### Genetic analysis

All patients underwent molecular analyses after written informed consent was obtained. Molecular analysis and variants evaluation were performed as recently described.^29^

### Pathology

Autopsy with detailed cardiac analysis was performed in accordance with current guidelines.^21^ Additional laboratory analyses (toxicology, chemistry, microbiology, and genetic testing) were performed. In patients accepting the invasive evaluation, when indicated, an endocardial 3-dimensional electroanatomic voltage mapping endomyocardial biopsy from the LV was performed to confirm diagnosis. For each patient, 3 to 5 samples were obtained for histology and immunohistochemistry, then fixed in 10% phosphate-buffered formalin (pH 7.35) and embedded in paraffin. Histological analysis was performed as previously described.^19^

### Primary outcomes

Patients were followed during regular outpatient clinical visits. The main endpoint was a combined arrhythmic endpoint (MAE), which included sudden cardiac death (SCD), aborted SCD, and appropriate ICD shock for ventricular tachycardia (VT)/ventricular fibrillation (VF). SCD was defined as an out-of-hospital death within 1 hour from symptom onset, in which non cardiac causes were excluded. Aborted SCD was defined as an appropriate ICD shock for ventricular arrhythmias, successful resuscitation following VF or spontaneous sustained VT causing hemodynamic compromise and requiring cardioversion.

### Statistical analysis

Continuous variables are reported as mean ± SD or median with lower and upper quartiles (Q1-Q3). The normality of the distribution of continuous variables was assessed with the Shapiro-Wilk test. Categorical variables are reported as frequencies and percentages. Comparisons between continuous variables were performed using the Student *t*-test or the Mann-Whitney *U* test, as appropriate. Comparisons between categorical variables were evaluated using the Fisher exact test or the Pearson chi-square test, as appropriate. Logistic regression was used to evaluate predictors of MAE occurring either as the first manifestation of ALVC or during follow-up and presented as the OR with 95% CI. The assumption of linearity between quantitative predictors and logit was verified as follows: each quantitative variable was transformed into a categorical variable according to quintiles and the median value of the variable in each quintile was used as the value for that category. Finally, a scatter plot was generated with the Logit in the y-axis and the quantitative variable categorized in the x-axis. Linearity was visually assessed in this scatter plot. Cox regression was used to evaluate predictors of MAE during follow-up, after exclusion of patients with MAE as the first manifestation of the disease with HR and 95% CI. The validity of the assumption of proportionality was verified by visual comparison of Cox and Kaplan-Meier curves and by analysis of interaction with time (Supplemental Table 1). The multivariable models were created as follows: starting with all variables that showed a statistically significant (*P* < 0.05) association with the effect in univariable analysis, a best subset regression procedure was used to identify the most suitable and parsimonious multivariable model based on the Akaike information criterion, which is an established parameter of the goodness of fit.

A two-tailed *P* value of < 0.05 was considered statistically significant. Statistics were performed using STATA 18.0/MP (StatCorp LLC).

## Results

### Study population

One hundred twenty-five patients with ALVC (64 men [51.2%], mean age 37 ± 15 years, range 10-75 years) were included in the study. Some data from 54 of these patients were part of the initial published series.^19^ Genetic test identified 119 pathogenic/likely PVs associated with ALVC and 2 variants of uncertain significance. *DSP* (MIM #125647) harbored the majority of genetic variants (81.5%) followed by plakophilin-2 (MIM# 602861, PKP2) (8.4%), *desmoglein-2* (MIM# 125671, DSG2) (6.7%), *plakoglobin* (MIM# 173325, JUP) (2.5%), and *desmocollin-2* (MIM# 125645, DSC2) (0.8%). The study included a patient who died suddenly with an autopsy diagnosis of ALVC and in whom genetic analysis was elusive. Myocardial biopsy was performed in 33 (24.4%) patients. Myocardial biopsy was positive in all the 5 patients with unperformed/inconclusive genetics. Twenty-three (18.4%) had an history of chest pain episodes with “hot phase” clinical presentation in 14 (11.2%). Four were diagnosed due to sustained VT and 13 due to aborted cardiac arrest. Of note, patients with variants on the PKP2 gene were in almost all cases symptomatic (9/10) with a very strong arrhythmic onset (2 patients had arrhythmic syncope, 3 had sustained VT and 3 patients had frequent PVC with palpitations). Table 1 shows the baseline clinical, structural, and genetic characteristics of our population.

**Table 1:** Baseline Clinical, Structural, Genetic, and Electrocardiographic Data of the Study Population (N = 125)

Age at diagnosis, y	37 ± 15
Male	64 (51.2)
Probands	89 (71.2)
Family history of AC/DCM	68 (54.4)
Family history of SCD	38 (30.4)
NYHA functional class I-II	121 (96.8)
NYHA functional class III	4 (3.2)
Atrial fibrillation	8 (6.4)
Unexplained syncope	15 (12.0)
NSVT	55 (44.0)
Cardiac magnetic resonance	
LVEDVi (mL/m^2^)	93.3 ± 22.9
LVEF, %	50.7 ± 10.1
LV WMA, %	80 (64.0)
RVEDVi (mL/m^2^)	84.3 ± 20.6
RVEF, %	53.1 ± 10.0
Intramyocardial fat signal	35 (28.0)
Segments with LGE	7 ± 4; 6 (4-10)
LGE pattern	
Ring-like	66 (52.8)
LGE distribution	
Subepicardial	95 (76.0)
Midmural	15 (12.0)
Transmural	15 (12.0)
Genetic testing	
Pathogenic/likely pathogenic variant	119/123 (96.7)
DSP	97/119 (81.5)
Non-DSP^a^	22/119 (18.5)
Electrocardiographic data	
Normal ECG	15 (12.0)
QRS (msec)	96 ± 15
First degree AV block	10 (8.0)
NSICD	2 (1.6)
RBBB	4 (3.2)
LAFB	15 (12.0)
LPFB	17 (13.6)
LBBB	1 (0.8)
Pathological Q waves	32 (25.6)
Lateral distribution	12 (9.6)
Inferior distribution	15 (12.0)
Precordial distribution	2 (1.6)
More 2 localizations	3 (2.4)
Fragmented QRS	46 (36.8)
Lateral distribution	6 (4.8)
Inferior distribution	28 (22.4)
Precordial distribution	2 (1.6)
More 2 localizations	10 (8.0)
Global LQRSV	12 (9.6)
LQRSV in limb leads	18 (14.4)
Local LQRSV	
Lateral distribution	29 (23.2)
Inferior distribution	19 (15.2)
Inferolateral distribution	5 (4.0)
Precordial and local distribution	12 (9.6)
Epsilon-like wave in inferior and/or lateral leads	12 (9.6)
QTc (ms)	409 ± 25
QTc ≥440 ms	10 (8.0)
Tzou criteria^b^	19 (15.2)
R-wave >3 mm V_1_	10 (8.0)
R/S ratio ≥0.5 in V_1_	33 (26.4)
R/S ratio ≥1 in V_1_	15 (12.0)
Bayés de Luna criteria^c^	7 (5.6)
TWI	58 (46.4)
Inferolateral TWI	9 (7.2)
Anterior TWI	11 (8.8)
Inferior TWI	5 (4.0)
Lateral TWI	11 (8.8)
Anterolateral TWI	15 (12.0)
Inferior-anterior-lateral TWI	7 (5.6)
New ECG criteria	
SV_1_ + RV_6_ ≤12 and RI + RII ≤8 (mm)	55 (44.0)

Values are mean ± SD, n (%), or median (Q1-Q3) as appropriate.

AC = arrhythmogenic cardiomyopathy; ALVC = arrhythmogenic left ventricular cardiomyopathy; AV = atrio-ventricular; DCM = dilated cardiomyopathy; DSP = desmoplakin; ECG = electrocardiogram; LAFB = left anterior fascicular block; LBBB = eft bundle branch block; LGE = late gadolinium enhancement; LPFB = left posterior fascicular block; LQRSV = low QRS voltage; LV = left ventricle; LVEDVi = left ventricular end-diastolic volume indexed; LVEF = left ventricular ejection fraction; NSICD = nonspecific intraventricular conduction delay; NSVT = nonsustained ventricular tachycardia; NYHA = New York Heart Association; QTc = corrected QT; RBBB = right bundle branch block; RVEDVi = right ventricular end-diastolic volume indexed; RVEF = right ventricular ejection fraction; SCD = sudden cardiac death; TWI = T-wave inversion; WMA = wall motion abnormalities.

a DSG2 (desmoglein-2) n = 8; JUP (plakoglobin) n = 3; PKP2 (plakophilin-2) n = 10; DSC2 (desmocollin-2) n = 1.

b V_1_R ≥0.15 mV and V_6_S ≥0.15 mV.

c R/S ratio in V_1_ ≥0.5 and R amplitude in V_1_ >3 mm.

### CMR data

At CMR evaluation, the mean left ventricular ejection fraction (LVEF) was 50.7% ± 10.1%, and the mean RVEF was 53.1% ± 10%. A mild RV disfunction was found in 27 patients (21.6%), 50% carriers of DSP mutations. LGE distribution was subepicardial and/or midmyocardial in 110 patients (88%) and transmural in 15 (12%). In patients with PKP2 PVs, a mild reduction of RVEF was found (mean RVEF 47% ± 5%) in 70% of cases with a mean LVEF of 56% ± 6%; LGE involved a median of 6 LV segments with a ring-like pattern in 4 patients. CMR data are summarized in Table 1.

### ECG findings

**Figure 1:**
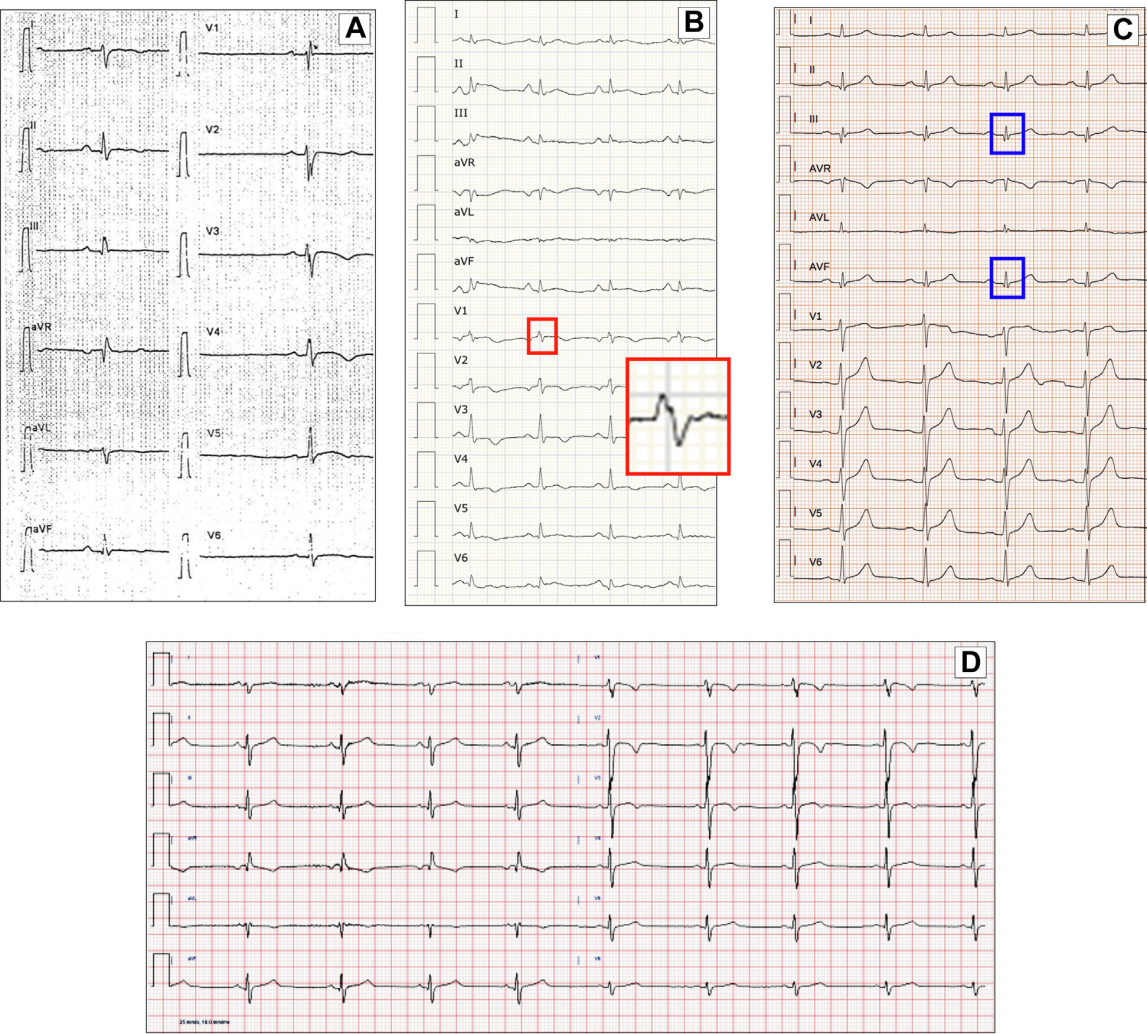
Electrocardiographic Findings in Patients With Arrhythmogenic Left Ventricular Cardiomyopathy (A) Electrocardiogram of patient #56 (19-year-old man, pathogenic variant in *desmoglein-2* c.271G>T, p.Gly91Ter) shows left posterior fascicular block, a fragmented QRS in V_1_ to V_3_ and T-wave inversion in V_3_ to V_5_. (B) Electrocardiogram of patient #83 (71-year-old man, likely pathogenic variant in *desmoglein-2* p.Val295Serfs∗6) displays global low QRS voltage, anterior T-wave inversion, and R/S ratio ≥0.5 in V_1_ (red box). (C) Electrocardiogram of patient #19 (35-year-old man with pathogenic variant in *desmoplakin* c.5210delG p.Gly1737AspfsTer16) displays low QRS voltage in limb leads and pathological inferior Q waves (blue boxes). (D) Patient #86 (14-year-old man, pathogenic variant in *plakophilin-2*, c.1378+1G>C), had an aborted sudden cardiac death at presentation; his electrocardiogram shows left posterior fascicular block and T-wave inversion in V_1_ to V_3_. A sum of the R-wave in I to II ≤8 mm and SV_1_ + RV_6_ ≤12 mm, a R/S ratio ≥0.5 in V_1_ are also present. All the electrocardiograms presented were performed at 25 mm/s with 1 mm/mV.

Electrocardiographic results are presented in Table 1 and in Figure 1. Among ECG parameters classically associated with ALVC, TWI was present in 46.4% of patients, LQRSV in limb leads in 14.4% of cases and epsilon-like waves in inferior and/or lateral leads in 9.6% of patients. Overall, any of these 3 ECG abnormalities was observed in 57.6% of patients. A fragmentation of QRS was found in 36.8% of patients. Among the recently described new ECG features, a RI + RII ≤8 mm and a SV1 + RV6 ≤12 mm was present in 44% of patients, a R/S ratio ≥0.5 in 26.4%, pathological Q waves in 25.6% of cases and LPFB in 13.6% of patients. Overall, these new ECG parameters were found in 61 (48.8%) patients, including 17 of those without the classical ECG signs.

### Relationship between genetic, CMR, and ECG findings

In patients with PVs in the *DSP* gene, both ring-like and nonring-like patterns were similarly represented. In contrast, patients with variants outside the *DSP* gene had a significantly higher frequency of nonring-like patterns (25.4% vs 10.6%, *P* = 0.026). These patients also had higher RV volumes, more reduced RVEF, more frequent transmural LGE distribution, and fewer affected segments, though not statistically significant. Supplemental Figure 1 illustrates the differences in LGE distribution between patients with DSP and patients without DSP. Regarding ECG findings, patients with variants outside the DSP gene were more likely to have LPFB (31.8 vs 7.2%, *P* < 0.001), pathological Q waves (50.0 vs 19.6%, *P* = 0.003), and an R/S ratio ≥0.5 in V_1_ (45.5 vs 20.6%, *P* = 0.02) (see Supplemental Table 2).

### Correlations between CMR and ECG findings

Several correlations between CMR and ECG findings were observed: 1) isolated anterior TWI was more frequent in patients with ring-like LGE (13.6% vs 3.4%, *P* = 0.045); 2) pathological Q waves (46.7% vs 22.7%, *P* = 0.046) and an R/S ratio ≥0.5 (46.7% vs 23.6%, *P* = 0.048) were more common in patients with transmural LGE; and 3) no patients with transmural LGE had a normal ECG. When comparing patients with normal and abnormal ECGs, those with normal ECGs had higher LVEF (58% ± 6% vs 50% ± 10%, *P* = 0.003). No significant differences were found in the number of LGE segments involved. Details on the relationship between clinical, genetic, ECG, and CMR findings are presented in Supplemental Table 3.

Patients with pathological Q waves in the inferior leads were more likely to show LGE in the lateral apical segment on CMR (60% vs 30.9%, *P* = 0.026). A higher involvement of the lateral, apical, and midanterolateral segments was observed in patients with pathological lateral Q-waves (85.3% vs 51.3%, *P* = 0.035). There were no significant differences in LGE distribution between patients with and without LPFB compared to the general population. However, it should be noted that 70% of patients with LPFB had inferior LGE. The presence of LGE in basal septal segments was more common in patients without an R/S ratio ≥0.5 in V_1_ compared to those with this finding (basal anterior interventricular septum 37% vs 9.1%, *P* = 0.003; basal inferior interventricular septum 38% vs 18.2%, *P* = 0.038). No significant differences in LGE distribution were found between patients with and without RI + RII ≤8 mm or a SV_1_ + RV_6_ ≤12 mm.

### Comparison of probands and relatives

Among 89 probands, 21 patients (23.6%) were asymptomatic at the first evaluation. The clinical suspicion in these patients was done by ECG abnormalities or by asymptomatic PVCs. Among symptomatic probands, 17 patients (19.1%) had a MAE or syncope as first clinical manifestation. The remaining probands referred palpitations or chest pain (with or without myocardial infarction with non-obstructive coronary arteries/myocarditis events).

In comparison with probands, relatives were less symptomatic (23.6% vs 58.3%; *P* < 0.001) and had more often a normal ECG (22.2% vs 7.9%; *P* = 0.026). No relatives showed global LQRSV, while a R/S ratio ≥0.5 in V_1_ was more prevalent in proband group (31.5% vs 13.9%, *P* = 0.044). At CMR, probands revealed more depressed LVEF (49.2% ± 10.3% vs 54.5% ± 8.6%, *P* = 0.007) and a major number of LGE segments involved (6 [Q1-Q3: 4-10] vs 4 [Q1-Q3: 3-7], *P* = 0.013). Details are reported in Supplemental Table 4.

### ECG progression during follow-up

**Figure 2:**
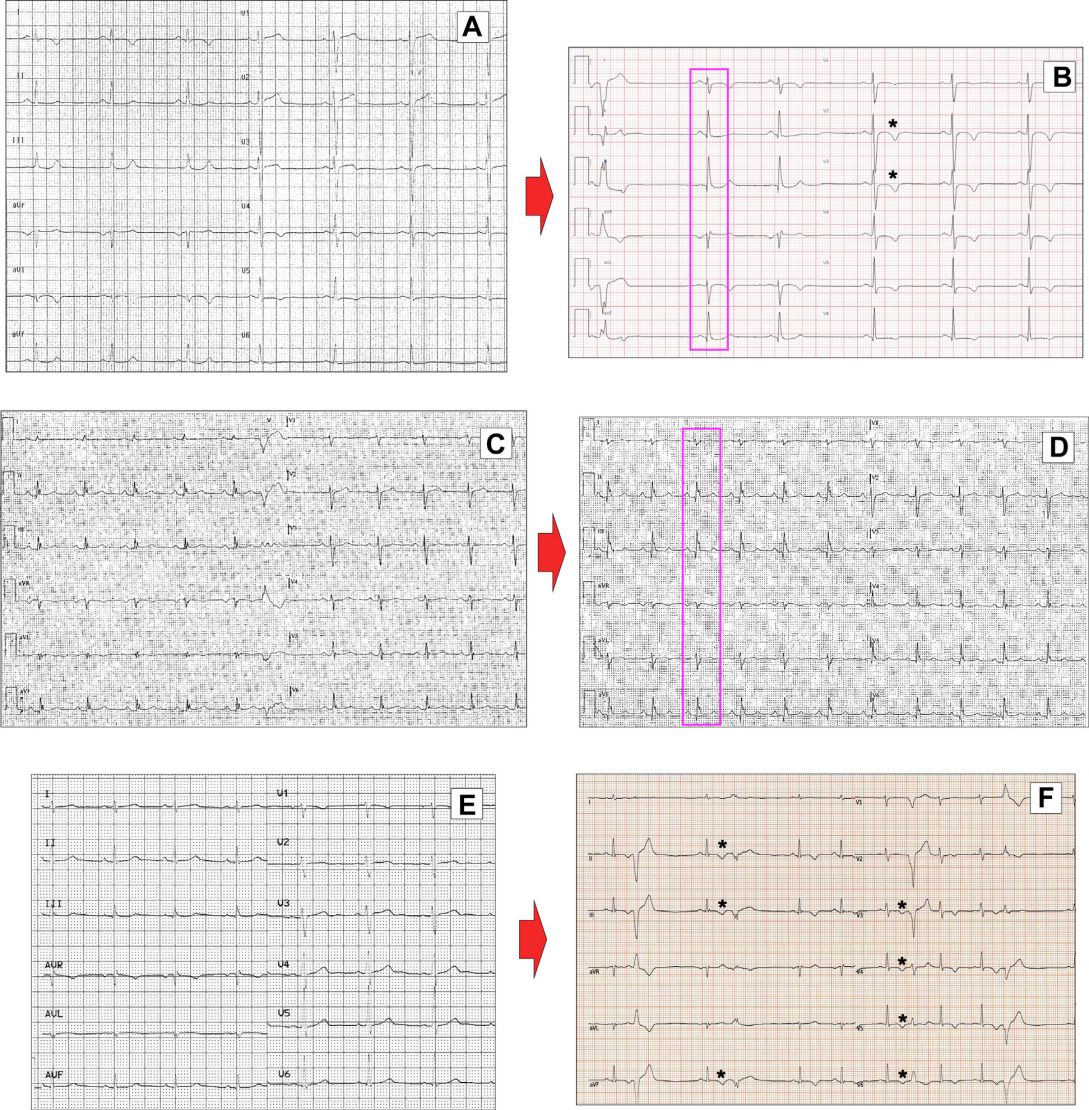
Electrocardiographic Changes During Follow-Up (A and B) Patient #38 is a man with a pathogenic variant in *desmoplakin* (deletion of the entire *desmoplakin* gene, 6p25.1-p24.3). Electrocardiogram at age 28 shows T-wave inversion in lateral leads. After 6 years, electrocardiogram shows the appearance of left posterior fascicular block (violet box) and T-wave inversion in leads V_2_ to V_3_ (asterisks). (C and D) Electrocardiograms of patient #45 (48-year-old man, likely pathogenic variant in *desmoplakin* c.860A>G, p.Asn287Ser) display the presence of low QRS voltages in lateral leads and the appearance over time of left posterior fascicular block and pathological Q waves in inferior leads (violet box). (E and F) Electrocardiogram of patient #108 (18-year-old man, pathogenic variant in *desmoplakin* c.2821C>T, P.Arg941∗) shows low QRS voltages in the lateral leads. After 4 years, the electrocardiogram shows the appearance of T-wave inversion in inferior leads and in V_3_ to V_6_ (asterisks), polymorphic premature ventricular beats.

Follow-up ECGs were available for analysis in 72 of 125 patients (57.6%). During a median follow-up of 45 months (Q1-Q3: 27-70), ECG changes were observed in 39/72 patients (Figure 2). Two of the 9 patients with normal baseline ECG developed ECG abnormalities during follow-up. New appearance or deepening of Q-waves, mainly in inferior leads, was detected in 8 patients. In 3 patients, we observed the new appearance of a LPFB. Low voltages in limb leads and in precordial leads (mainly in V_5_-V_6_) was detected in 15 and 23 patients, respectively. Ventricular repolarization abnormalities were noted in 17 subjects with occurrence of TWI in 10.

### Follow-up

The median follow-up was 57 months (Q1-Q3: 25-89). Sixty-seven (53.6%) patients received an ICD (43 primary prevention, 24 secondary prevention). MAE occurred in 35 patients (28%) (SCD = 3, aborted cardiac arrest = 21, ICD shock for VT/VF = 11): 17 patients had a MAE as the first manifestation of the disease and 23 patients had a MAE during follow-up. Among those with the MAE as the first manifestation of the disease, 5 had recurrent MAE during follow-up. Table 2 shows the main clinical, structural, genetic, and ECG findings of the study population according to presence or absence of composite endpoint. MAE were significantly more frequent in patients with unexplained syncope, transmural LGE, and reduced LVEF and RVEF. In addition, patients with variants not occurring in the DSP gene experienced more MAE (54.5% vs 19.6%; *P* = 0.001).

**Table 2:** Baseline Clinical, Structural, Genetic, and ECG Characteristics of the Study Population According to Occurrence of Major Arrhythmic Events

	Major Arrhythmic Events (n = 35)	No Major Arrhythmic Events (n = 90)	*P* Value
Age at diagnosis, y	40 ± 15	36 ± 15	0.48
Male	22 (62.9)	42 (46.7)	0.11
Proband	31 (88.6)	58 (64.4)	**0.052**
Family history of DCM/AC	12 (34.3)	56 (62.2)	**0.005**
Family history of SCD	7 (20.0)	31 (34.4)	0.13
NYHA functional class I-II	34 (97.1)	87 (96.7)	0.70
NYHA functional class III	1 (2.8)	3 (3.3)	0.98
Atrial fibrillation	3 (8.6)	5 (5.6)	0.42
Unexplained syncope	13 (37.1)	2 (2.2)	**<0.001**
NSVT	16 (45.7)	39 (43.3)	0.67
Asymptomatic	7 (20.0)	28 (31.1)	0.21
Cardiac magnetic resonance			
LVEDVi (mL/m^2^)	91.0 ± 21.5	94.2 ± 23.4	0.96
LVEF, %	46.6 ± 9.1	52.3 ± 10.0	**0.010**
LVEF <50%	22 (62.9)	30 (33.3)	0.55
RVEDVi (mL/m^2^)	89.3 ± 24.6	82.5 ± 18.8	0.22
RVEF, %	48.4 ± 12.1	54.8 ± 8.5	**0.021**
Segments with LGE	7 ± 4; 6 (3-11)	7 ± 4; 6 (4-8)	>0.99
1-3 segments	9 (25.7)	17 (18.9)	0.31
4-6 segments	11 (31.4)	35 (38.9)	0.24
>6 segments	15 (42.8)	38 (42.2)	0.89
LGE pattern			
Ring-like	21 (60.0)	45 (50.0)	0.31
LGE distribution			
Subepicardial	24 (68.6)	71 (78.9)	0.11
Midmural	1 (2.8)	14 (15.6)	**0.048**
Transmural	10 (28.6)	5 (5.6)	**0.005**
Genetic testing			
Pathogenic/likely pathogenic variant	31/33 (93.9)	88/90 (98.9)	0.10
DSP	19/31 (61.3)	78/88 (88.6)	**0.003**
Non-DSP^a^	12/31 (38.7)	10/88 (11.4)	**0.003**
ECG			
Normal ECG	1 (2.9)	14 (15.6)	0.051
QRS (msec)	99 ± 17	95 ± 14	**0.004**
First degree AV block	4 (11.4)	6 (6.7)	0.32
NSICD	0	2 (2.2)	0.65
RBBB	2 (5.7)	2 (2.2)	0.15
LAFB	6 (17.1)	9 (10.0)	0.15
LPFB	10 (28.6)	7 (7.8)	**<0.001**
LBBB	1 (2.9)	0	0.11
Pathological Q waves	9 (25.7)	23 (25.6)	0.99
Lateral distribution	4 (11.4)	8 (8.9)	0.64
Inferior distribution	4 (11.4)	11 (12.2)	0.89
Precordial distribution	0	2 (2.2)	0.65
More 2 localizations	1 (2.9)	2 (2.2)	0.98
Fragmented QRS	12 (34.3)	34 (37.8)	0.56
Lateral distribution	2 (5.7)	4 (4.4)	0.52
Inferior distribution	6 (17.1)	22 (24.4)	0.22
Precordial distribution	1 (2.9)	1 (1.1)	0.31
More 2 localizations	3 (8.6)	7 (7.8)	0.80
Global LQRSV	6 (17.1)	6 (6.7)	0.091
LQRSV in limb leads	2 (5.7)	16 (17.8)	0.089
Local LQRSV			
Lateral distribution	8 (22.9)	21 (23.3)	0.89
Inferior distribution	5 (14.3)	14 (15.6)	0.84
Inferolateral distribution	2 (5.7)	3 (3.3)	0.31
Precordial and local distribution	2 (5.7)	10 (11.1)	0.20
QTc (msec)	411 ± 27	409 ± 24	0.92
QTc ≥440 ms	5 (14.3)	5 (5.6)	0.11
Tzou criteria^b^	7 (20.0)	12 (13.3)	0.18
R >3 mm V_1_	4 (11.4)	6 (6.7)	0.32
R/S ratio ≥0.5 in V_1_	15 (42.9)	18 (20.0)	**0.009**
R/S ratio ≥1 in V_1_	8 (22.9)	7 (7.8)	**0.021**
Bayés de Luna criteria^c^	4 (11.4)	3 (3.3)	0.071
TWI	21 (60.0)	37 (41.1)	0.052
Inferolateral TWI	4 (11.4)	5 (5.6)	0.34
Anterior TWI	6 (17.1)	5 (5.6)	0.055
Inferior TWI	1 (2.9)	4 (4.4)	0.70
Lateral TWI	2 (5.7)	9 (10.0)	0.30
Anterolateral TWI	6 (17.1)	9 (10.0)	0.15
Inferior-anterior-lateral TWI	2 (5.7)	5 (5.6)	098
New ECG criteria			
SV_1_ + RV_6_ ≤12 and RI + RII ≤8 (mm)	22 (62.9)	33 (36.7)	**0.008**

Values are mean ± SD, n (%), or median (Q1-Q3) as. appropriate. **Bold** values denote statistical significance at the *P* < 0.05 level.

Abbreviations as in Table 1.

a DSG2 (desmoglein-2) n = 8; JUP (plakoglobin) n = 3; PKP2 (plakophilin-2) n = 10; DSC2 (desmocollin-2) n = 1].

b V_1_R ≥0.15 mV and V_6_S ≥0.15 mV.

c R/S ratio in V_1_ ≥0.5 and R amplitude in V_1_ >3 mm.

Among ECG findings, LPFB, R/S ratio ≥0.5 and ≥1 in V1 were significantly more frequent in patients with MAE. Of note, the association between LPFB and MAE was limited to patients without DSP while among carriers of DSP variants there was no difference in the prevalence of MAE in those with or without LPFB (*P* > 0.99); a significant linear increase (*P* < 0.001) in the prevalence MAE was observed going from patients without DSP with or without LPFB (20% with MAE) to patients with DSP without LPFB (40% with MAE) and patients with DSP with LPFB (86% with MAE). Only 1 of the 15 patients with a normal ECG experienced MAE in comparison with 34/110 patients (30.1%) with an abnormal ECG (*P* = 0.057).

The univariable and multivariable logistic regression analyses for MAE are shown in Table 3. The univariable analysis revealed that, among ECG variables, LPFB (OR: 4.74; 95% CI: 1.6-13.8; *P* = 0.004), R/S ratio ≥0.5 in lead V1 (OR: 3.00; 95% CI: 1.3-7.0; *P* = 0.011), anterior TWI (OR: 2.54; 95% CI: 1.1-6.0; *P* = 0.035), and SV1 + RV6 ≤12 mm and RI + RII ≤8 mm (OR: 2.92; 95% CI: 1.3-6.6; *P* = 0.009) were significant predictors for the composite outcome. However, in the multivariable analysis including these 4 variables, only LPFB remained a significant predictor (OR: 1.2; *P* = 0.04). In addition to ECG parameters, syncope, transmural LGE, non-DSP variants, LVEF, and RVEF showed significant association with MAE in univariable analysis (Table 3, Central Illustration).

**Table 3:** Univariable and Multivariable Logistic Regressions of MAE With ECG Parameters, Syncope, Cardiac Magnetic Resonance, and Genetic Variables

Variable	Univariable OR (CI)	*P* Value	Multivariable OR (CI)	*P* Value
ECG Variables				
QRS (ms)	1.02 (0.99-1.04)	0.21		
RBBB	2.67 (0.36-19.71)	0.34		
LAFB	1.86 (0.61-5.69)	0.28		
LPFB	4.74 (1.64-13.75)	0.004		
LBBB	–			
Pathological Q waves	1.07 (0.44-2.62)	0.88		
Global LQRSV	2.90 (0.87-9.69)	0.084		
LQRSV in limb leads	0.28 (0.06-1.29)	0.10		
R/S ratio ≥0.5 in V_1_	3.00 (1.29-7.00)	0.011		
TWI	2.15 (0.97-4.76)	0.06		
Anterior TWI	2.54 (1.07-6.03)	0.035		
SV_1_ + RV_6_ ≤12 and RI + RII ≤8 (mm)	2.92 (1.30-6.56)	0.009		
Clinical, structural, and genetic variables				
Syncope	26.00 (5.46-123.76)	<0.001		
Transmural LGE	6.80 (2.13-21.74)	0.001		
LVEF, %	0.94 (0.91-0.98)	0.006		
RVEF, %	0.94 (0.90-0.98)	0.002		
Non-DSP	4.93 (1.85-13.09)	0.001		
Best multivariable model (AUC: 0.9; 95% CI: 0.83-0.95)				
LPFB			4.7 (1.2-18.3)	0.03
Syncope			84.95 (14-496)	<0.001
Transmural LGE			9.95 (2.3-36)	0.002
RVEF, %			0.92 (0.87-0.97)	0.003

Values are odds ratio (OR) (95% confidence interval, CI).

AUC = area under the curve; MAE = major arrhythmic event; other abbreviations as in Table 1.

**Central Illustration:**
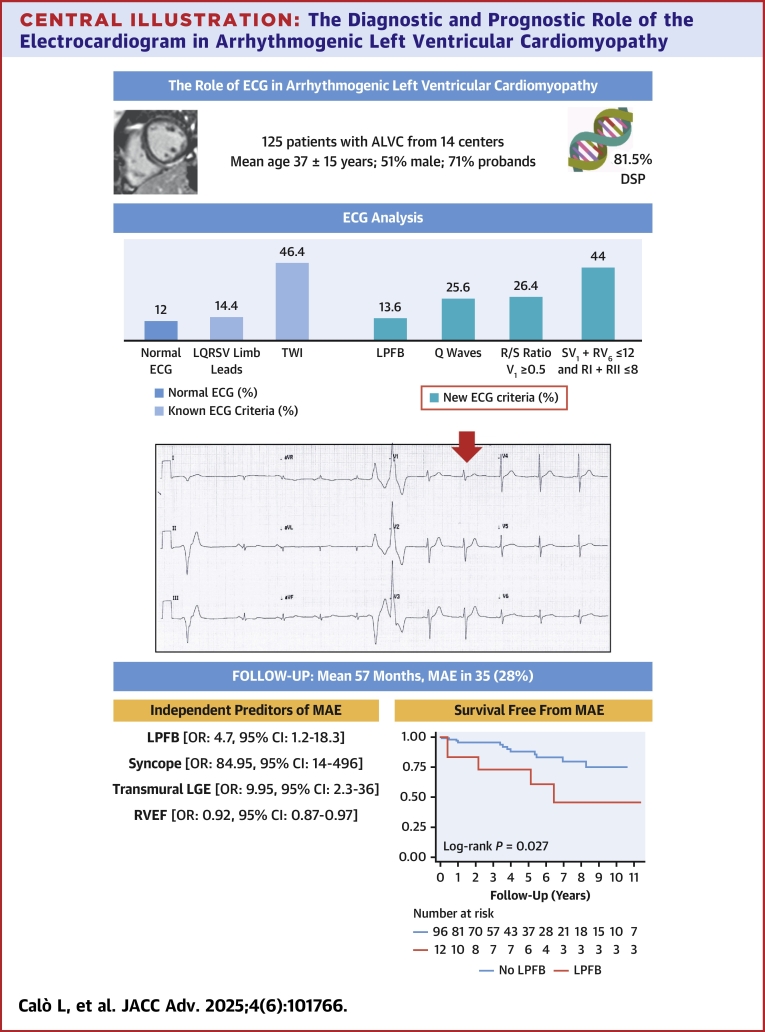
The Diagnostic and Prognostic Role of the Electrocardiogram in Arrhythmogenic Left Ventricular Cardiomyopathy ALVC = arrhythmogenic left ventricular cardiomyopathy; DSP = desmoplakin; ECG = electrocardiogram; LGE = late gadolinium enhancement; LPFB = left posterior fascicular block; LQRSV = low QRS voltages; MAE = major arrhythmic events; RVEF = right ventricular ejection fraction; TWI = T-wave inversion.

In the multivariable analysis, LPFB (OR: 4.7; 95% CI: 1.2-18.3; *P* = 0.03), syncope (OR: 84.95; 95% CI: 14-496; *P* < 0.001), transmural LGE (OR: 9.95; 95% CI: 2.3-36; *P* = 0.002), and RVEF (OR: 0.92; 95% CI: 0.87-0.97; *P* = 0.003) were the independent predictors of MAE. The multivariable model including these 4 variables achieved an excellent predictive ability (area under the curve: 0.9; 95% CI: 0.83-0.95). Of note, non-DSP variants no longer had a statistically significant association with MAE after adjustment for transmural LGE and RVEF (adjusted *P* = 0.09).

To evaluate a primary prevention scenario, we excluded the 17 patients with MAE at the time of diagnosis. As shown in Supplemental Table 5, among ECG parameters, only LPFB was a significant predictor of MAE at follow-up (HR: 3.1; 95% CI: 1.1-8.7; *P* = 0.036). The association between LPFB and MAE is also graphically represented in the Kaplan-Meier curves of Supplemental Figure 2 and Central Illustration. In multivariable Cox analysis, including also clinical, imaging, and genetic variables, LPFB remained an independent predictor of MAE (HR: 3.98; 95% CI: 1.3-12.0; *P* = 0.014) together with syncope (HR: 19.13; 95% CI: 5.8-63.0; *P* < 0.001) and transmural LGE (HR: 10.57; 95% CI: 2.9-38.0; *P* < 0.001). A model including these 3 variables achieved good prognostic performance (Harrells' C = 0.79; 95% CI: 0.66-0.89).

## Discussion

### Main Findings

The following main results were observed. Patients with ALVC exhibit distinct ECG characteristics. Some of these ECG signs are new or little known such as pathological Q-waves, LPFB, a R/S ratio in V_1_ ≥0.5, a sum of the R-wave in I to II ≤8 mm, and S-wave in V_1_ plus R-wave in V_6_ ≤12 mm. Moreover, the ECG showed important changes over time. We observed specific correlations between ECG, CMR, and genetic findings. Among ECG variables, LPFB was the only one which remained an independent predictor of MAE at multivariable analysis. A risk-stratification model including LPFB, syncope, transmural LGE, and RVEF achieved excellent predictive ability for MAE (Central Illustration).

### ECG findings in ALVC

Normal ECG was present in 12% of patients but in none of those with transmural LGE. Furthermore, the prevalence of normal ECG was significantly higher in relatives than in probands.

LQRSV in limb leads and TWI in V_5_ to V_6_ were included among the ECG criteria for diagnosis of ALVC in a recent consensus document.^11^ However, these ECG parameters are considered a minor criterion because of their low specificity.^11^

In agreement with previous studies,^1^, ^2^, ^3^, ^4^, ^5^, ^6^, ^7^, ^8^^,^^14^^,^^16^ we commonly observed TWI; actually, TWI was the most frequent ECG abnormality. LQRSV in limb leads, in line with the data of literature, was observed in a minority of patients (14%).

Epsilon-like waves in inferior and/or lateral leads were rarely found, as previously described.^4^^,^^6^

We detected fQRS in about one-third of patients, but certainly QRS fragmentation may be missed with lower filter settings such as 40 to 60 Hz. In fact, accurate recording of fQRS on a 12-lead ECG requires an optimal low-pass filter setting (100-150 Hz).

We have recently described new peculiar ECG signs in ALVC: abnormal Q waves, LPFB, and a prominent R-wave in V_1_ with a R/S ratio ≥0.5.^19^ Furthermore, we have found LQRSV in inferolateral leads in comparison with controls, and that the sum of the R-wave ≤8 mm in leads I to II and the sum of the S-wave in V_1_ and R-wave in V_6_ ≤12 mm were very specific criteria for ALVC with a sensitivity of 44.4%.^19^ These newly described ECG signs were confirmed to be frequent in the present study.

Abnormal Q-waves were present in over a quarter of our patients, specifically in the inferolateral leads and more rarely in the precordial leads; in the majority of cases such Q-waves corresponded to the presence of lateral (and mostly latero-apical) LGE. A prominent R in V_1_ with a R/S ratio ≥0.5, related to loss of the LV basal-lateral activation forces^26^^,^^27^ was confirmed to be frequent (26.4%). This ECG sign, almost never described in previous reports, is not an uncommon occurrence in ALVC and often goes unnoticed.^19^

A LPFB was present in 17 patients and in isolation in 3 cases. Notably, patients with a non-DSP genotypes presented more frequently a LPFB. Given its frequent association with inferior LGE, LPFB may be an expression of fibrotic remodeling of the inferior/inferoseptal wall, which damages the posterior radiation of the left bundle branch.

LPFB may be underdiagnosed, and it is often not described in figures presenting ECGs of studies on ALVC, where it is clearly visible.^19^ On the other hand, it is important not to overdiagnose LPFB. In fact, numerous causes of right QRS axis deviation are not related to LPFB including young age, vertical heart or RV hypertrophy. Therefore, despite strict ECG criteria, the diagnosis of LPFB should necessarily involve a combined clinical-ECG approach.^23^ The sum of the R-wave ≤8 mm in leads I to II and the S-wave in V_1_ and R-wave in V_6_ ≤12 mm was simultaneously present in about half of our patients. This finding confirms the importance of observing not only the low voltage in all leads or exclusively in limb leads, as observed many years ago by Sokolow and Lyon.^30^^,^^31^

Of great interest is the fact that the ECG changes over time in about one-half of the patients. Therefore, ECG could be a marker of disease progression and maybe fibrosis evolution during follow-up. However, to confirm this hypothesis, we would need specific studies with repeated CMR during follow-up.

### Relationship between ECG signs, CMR, and genetic findings

We observed a ring-like pattern in about half of the cases.

Confirming recent observations,^4^^,^^14^, ^15^, ^16^^,^^18^ we detected a *DSP* pathogenic/likely PVs in the vast majority (81.5%) of patients.

In arrhythmogenic cardiomyopathy, it has been noted that the nondesmosomal mutations more frequently show a subepicardial LGE pattern, whereas the desmosomal mutations has a greater prevalence of a ring-like pattern.^17^

In our cohort, transmural LGE was present in a minority (12%) of patients, but its prevalence was significantly higher (32%) in the variants not located within the DSP gene. The non-DSP group exhibited a higher frequency of pathological Q-waves, LPFB, R/S ratio in V_1_ ≥0.5, and TWI. This can be explained by the prevalent presence of transmural LGE for Q-waves, R/S ratio in V_1_ ≥0.5, and TWI.

### Prognostic implications

Our study provides further confirmation of the unfavorable arrhythmic prognosis observed in patients with ALVC, since at least 1 MAE occurred in 28% of cases.

To our knowledge, this is the first study that combines clinical, imaging, genetic, and ECG variables to improve the risk prediction of MAE in ALVC.

An R/S ratio ≥0.5 in lead V_1_, anterior TWI, and SV_1_ + RV_6_ ≤12 mm and RI + RII ≤8 mm were associated with MAE in the univariable analysis; however, this association was lost when adjusting for other covariates. Since ECG is a simple and accessible initial test, these parameters could help to define the arrhythmic risk of a single patient at the initial visit, while waiting for imaging and genetic data.

Among ECG variables, LPFB was indeed the strongest predictor of MAE and the only one which continued to be significant after adjustment for clinical, CMR, and genetic variables. In addition, after excluding patients with a MAE as the first manifestation of ALVC (primary prevention scenario), LPFB was still a significant, independent, and strong predictor of MAE, increasing the risk 4-fold after adjustment for syncope and transmural LGE.

Recently, we described how LPFB, a very rare finding in the general population,^23^ was detected in about 9% of young SCD/aborted cardiac arrest patients.^32^ Another study in a large Danish registry confirmed that LPFB was associated with the highest risk of death (HR: 2.09), although they were in the youngest age group (median age: 35 years).^33^

Besides the association between LPFB and inferior/inferoseptal scar, the arrhythmogenicity of LPFB may also depend on the involvement of the Purkinje system within the scar. It is well known that Purkinje fibers are a major source of ventricular arrhythmias, both monomorphic VT and VF.^34^

Indeed, one of the most relevant clinical messages of this manuscript is that LPFB should be regarded as a high-risk feature in patients with ALVC.

The other independent predictors of MAE were syncope, transmural LGE, and RVEF in line with prior evidence from the literature.^13^, ^14^, ^15^, ^16^^,^^35^

In a population of patients with apparently idiopathic nonsustained ventricular arrhythmias, including a majority of patients without LGE, the presence of ring-like LGE (present in only 4% of those patients) was an independent predictor of ventricular arrhythmias or sudden death at follow-up.^36^ In that cohort, ring-like LGE likely identified patients with probable ALVC, and it is expected that those patients will have a worse arrhythmic prognosis as compared to patients without LGE or without structural heart disease at all. By contrast, in a homogeneous cohort of patients with ALVC, where the overall arrhythmic risk was already high, we observed that ring-like LGE was not associated with a significant increase in the arrhythmic risk. This finding should be confirmed in further studies focused on ALVC. Transmural LGE was the scar-related parameter which maintained a significant association with MAE in multivariable analysis. In addition, transmural LGE was an independent predictor of MAE during follow-up (after excluding patients with MAE as the first manifestation of the disease). In this primary prevention scenario, transmural LGE increased the risk of MAE more than 10-fold. These findings are in line with a prior report indicating that transmural LGE is an independent predictor of ventricular arrhythmias and sudden death in nonischemic DCM.^37^

The prognostic role of pathogenic/likely PVs in different desmosomal genes in patients with ALVC has not been specifically evaluated. Some *DSP* variants, particularly truncating mutations, have been associated with a higher incidence of SCD.^15^^,^^38^ Sporadic instances of pathogenic mutations in the PKP2 gene have been described, displaying a high-risk phenotype, especially when combined with other pathogenic mutation of desmosomal and nondesmosomal genotypes.^39^^,^^40^ Additionally, reported cases of ALVC with *DSG2* and *JUP* mutations have shown a high risk of SCD.^4^^,^^39^ For the first time, we have showed that carriers of variants not located in the DSP gene have significantly higher arrhythmic risk as compared to carriers of DSP variants. However, such differences in outcomes seems to be related to specific clinical features of non-DSP carriers, such as lower RVEF and higher prevalence of LPFB and transmural LGE. The prognostic role of pathogenic/likely PVs in different desmosomal genes should be further evaluated in future studies.

The predictive model for MAE including syncope, LPFB, transmural LGE, and RVEF achieved a very high predictive ability (area under the curve: 0.9). This finding has indeed a great clinical relevance as it may help in the risk stratification of these patients. In addition, this observation may be important for future studies in the field, which could attempt to validate our results and further improve risk prediction.

### Study limitations

This was a retrospective multicenter study with consequent limitations. The strict inclusion criteria aimed to identify a well-selected cohort, reducing the number of enrolled patients. The rarity of the disease leads to a relatively small sample size that limits the ability of our models to discriminate (eg, wide CIs). ECGs were performed at different centers with different filters, which could alter visualization of QRS fragmentation/notching or epsilon-waves. The population includes an overwhelming proportion of DSP carriers which does not necessarily reflect the overall ALVC landscape.

A potential limitation is the definition of ALVC. ALVC currently does not have widely accepted diagnostic criteria. Recently, the European Society of Cardiology 2023 guidelines on cardiomyopathies^41^ proposed the term nondilated left ventricular cardiomyopathy that included patients that up until now may have variably been described as having DCM (but without LV dilatation), ALVC, Arrhythmogenic Right Ventricular Cardiomyopathy (ARVC), or arrhythmogenic DCM (but often without fulfilling diagnostic criteria for ARVC). Therefore, it is a phenotypic entity that has been described in the past and it overlaps with ARVC, DCM, and nondilated left ventricular cardiomyopathy phenotypes. In this study, planned over 4 years ago, we considered a diagnosis of ALVC based on morpho-functional criteria and restricted the genetic defect to desmosomal genes only in order to have a more homogeneous study group.

Another limitation is related to the absence of a centralized CMR analysis. However, all CMR were evaluated by experienced readers with either Society for Cardiovascular Magnetic Resonance or European Association of Cardiovascular Imaging Level II or III accreditation status; in addition, cardiac volumes, function, and LGE were analyzed in accordance with corrent guidelines.^42^

Logistic and Cox regression covariates have relatively wide CIs, especially for syncope. This implies a limited precision in the assessment of the true strength of the association with the outcome, without affecting its statistical significance.

Furthermore, we have not systematically examined the ECG and the CMR during follow-up, since data were not always available. Therefore, the risk of MAE could have a tendency to modify over time as patients develop progressive LV fibrosis, and the variables we analyzed at baseline stratification might require restratification as they have changed.

## Conclusions

ECG presents peculiar findings in ALVC, some of which are well-known, such as TWI and LQRSV in limb leads, and other less known, such as LPFB, pathological Q waves, R/S ratio ≥0.5 in V_1_, and local LQRSV in inferior and lateral leads.

Among ECG parameters, LPFB was the only one which retained a significant association with MAEs in multivariable analysis, after adjustment for clinical and CMR variables. A model including LPFB, syncope, transmural LGE, and RVEF achieved an excellent predictive ability for MAEs.

Perspectives **COMPETENCY IN MEDICAL KNOWLEDGE:** In line with prior observations, we confirm that patients with ALVC have a very high risk of ventricular arrhythmias and sudden death. ECG analysis remains a key element in the evaluation of patients with ALVC. Recognition of some new ECG signs, on top of classical signs such as T-wave inversion and low QRS voltage in limb leads, can help in early diagnosis and risk stratification in these patients. Among the ECG parameters, LPFB emerges as noteworthy predictor of ventricular arrhythmias or sudden death also in a primary prevention scenario, increasing the risk 4-fold.

**TRANSLATIONAL OUTLOOK:** Future studies in the field could attempt to validate our results and further improve risk prediction.

## Funding Support and Author Disclosures

The work reported in this publication was funded by the 10.13039/501100003196Italian Ministry of Health, RC-2022-2773270 project to Dr Biagini and by FSC 2014 to 2020, grant id. T3-AN-04 “GENERA” to Dr Novelli. All other authors have reported that they have no relationships relevant to the contents of this paper to disclose.
