# Supplementary material for: The Diagnostic and Prognostic Value of the 12-Lead ECG in Arrhythmogenic Left Ventricular Cardiomyopathy
Source: JACC Adv. 2025 May 12;4(6):101766. doi: 10.1016/j.jacadv.2025.101766 (PMC12141911; doi:10.1016/j.jacadv.2025.101766)

SUPPLEMENTAL TABLES AND FIGURES

Supplemental Table 1. Evaluation of the interaction with time of each variable evaluated for its association with the primary endpoint.

	p
**LPFB**	0.51
**R/S ratio ≥0.5 in V1**	0.63
**Anterior TWI**	0.16
**SV1+RV6 ≤12 and RI+RII ≤8 (mm)**	0.18
**Syncope**	0.28
**Transmural LGE**	0.93
**LVEF, %**	0.44
**RVEF, %**	0.91
**Non-DSP**	0.10

P value is obtained with the Wald's method.
DSP, desmoplakin; LGE, late gadolinium enhancement; LPFB, Left posterior fascicular block; LVEF, left ventricular ejection fraction; RVEDVi, right ventricular end-diastolic volume indexed; RVEF, right ventricular ejection fraction; TWI, T wave inversion.

Supplemental Table 2. Clinical, structural and electrocardiographic characteristics of the study population according to the genotype group.

	**DSP genotypes** (n=97)	**Non-DSP genotypes** (n=22)	**P Value**
Age at diagnosis, years	36±15	36±20	> 0.99
Male	42 (43.3)	16 (72.7)	**0.011**
Family history of AC/DCM	60 (61.9)	6 (27.3)	**0.003**
Unexplained syncope	10 (10.3)	4 (18.2)	0.29
NSVT	38 (39.2)	13 (59.1)	0.088
MAE	19 (19.6)	12 (54.5)	**0.001**
ECG			
**Normal ECG**	13 (13.4)	2 (9.1)	0.58
**QRS (ms)**	95±14	99±17	0.80
**First degree AV block**	8 (8.2)	0	0.17
**NSICD**	1 (1.0)	0	0.63
**RBBB**	0	3 (13.6)	**0.0002**
**LAFB**	12 (12.4)	2 (9.1)	0.49
**LPFB**	7 (7.2)	7 (31.8)	**<0.001**
**LBBB**	1 (1.0)	0	0.63
**Pathological Q waves**	19 (19.6)	11 (50.0)	**0.003**
Lateral distribution	6 (6.2)	5 (22.7)	**0.013**
Inferior distribution	10 (10.3)	5 (22.7)	0.12
Precordial distribution	2 (2.1)	0	0.50
More 2 localizations	1 (1.0)	1 (4.5)	0.39
**Fragmented QRS**	38 (39.2)	5 (22.7)	0.16
Lateral distribution	5 (5.2)	1 (4.5)	0.73
Inferior distribution	26 (26.8)	0	**<0.001**
Precordial distribution	1 (1.0)	1 (4.5)	0.39
More 2 localizations	6 (6.2)	3 (13.6)	0.13
**Global LQRSV**	7 (7.2)	5 (22.7)	**0.024**
**LQRSV in limb leads**	15 (15.5)	2 (9.1)	0.46
**Local LQRSV**			
Lateral distribution	23 (23.7)	5 (22.7)	0.89
Inferior distribution	17 (17.5)	1 (4.5)	0.12
Inferolateral distribution	4 (4.1)	0	0.17
Precordial and local	10 (10.3)	2 (9.1)	0.87
**QTc (msec)**	408±23	419±28	0.054
**QTc ≥440 msec**	6 (6.2)	4 (18.2)	0.065
**Tzou criteria***	13 (13.4)	3 (13.6)	0.98
**R >3 mm V1**	6 (6.2)	3 (13.6)	0.20
**R/S ratio ≥0.5 in V1**	20 (20.6)	10 (45.5)	**0.020**

**R/S ratio ≥1 in V1**	8 (8.2)	6 (27.3)	**0.012**
**Bayés de Luna criteria †**	4 (4.1)	2 (9.1)	0.33
**TWI**	37 (38.1)	16 (72.7)	**0.003**
Inferolateral TWI	5 (5.2)	2 (9.1)	0.27
Anterior TWI	3 (3.1)	7 (31.8)	**<0.001**
Inferior TWI	5 (5.2)	0	0.40
Lateral TWI	8 (8.2)	1 (4.5)	0.57
Anterolateral TWI	12 (12.4)	3 (13.6)	0.98
Inferior-anterior-lateral TWI	4 (4.1)	2 (9.1)	0.15
**NEW ECG CRITERIA**			
SV1+RV6 ≤12 and RI + RII ≤8 (mm)	38 (39.2)	14 (63.6)	**0.038**
Cardiac magnetic resonance			
LVEDVi (ml/m2)	93.7±21.9	84.8±19.6	0.082
LVEF, %	50.7±10.3	52.6±8.3	0.78
RVEDVi (ml/m2)	79.8±18.1	105.2±20.7	**<0.001**
RVEF, %	55.2±8.2	44.0±12.0	**<0.001**
Segments with LGE	7±4; 6 (4-10)	5±4; 4 (3-7)	0.080
LGE pattern			
- Ringlike	54 (55.7)	7 (31.8)	**0.042**
LGE distribution			
- Subepicardial	78 (80.4)	13 (59.1)	**0.037**
- Midmural	12 (12.4)	2 (9.1)	0.49
- Transmural	7 (7.2)	7 (31.8)	**0.001**

Data are expressed as n (%) or mean ± standard deviation or median (Q1-Q3) as appropriate.
AC indicates Arrhythmogenic Cardiomyopathy; AV, atrio-ventricular; DCM, dilated cardiomyopathy; DSP, desmoplakin; LAFB, Left anterior fascicular block; LBBB, Left bundle branch block; LGE, late gadolinium enhancement; LPFB, Left posterior fascicular block; LQRSV, low QRS voltages; LVEDVi, left ventricular end-diastolic volume indexed; LVEF, left ventricular ejection fraction; MAE, Major Arrhythmic Events; NSICD, Non-specific intraventricular conduction delay; NSVT, non sustained ventricular tachycardia; RBBB, Right bundle branch block; RVEDVi, right ventricular end-diastolic volume indexed; RVEF, right ventricular ejection fraction; TWI, T wave inversion. * V1R ≥ 0.15 mV and V6S ≥ 0.15 mV
† R/S ratio in V1 ≥ 0.5 and R amplitude in V1 >3 mm

Supplemental Table 3. Relationship between main clinical, genetic, electrocardiographic findings and pattern/distribution of LV scar.

	**Ring like pattern** (n=66)	**Non-Ringlike pattern** (n=59)	**P Value**	**Transmural distribution** (n=15)	**Non-transmural distribution** (n=110)	**P Value**
Age at diagnosis, years	35±15	39±16	0.45	42±20	36±15	0.42
Male	27 (40.9)	37 (62.7)	**0.014**	10 (66.7)	54 (49.1)	0.61
Family history of AC/DCM	32 (48.5)	36 (61.0)	0.18	5 (33.3)	63 (57.3)	0.081
Unexplained syncope	9 (13.6)	6 (10.2)	0.38	0	15 (13.6)	0.15
NSVT	34 (51.5)	21 (35.6)	0.093	4 (26.7)	51 (46.4)	0.15
MAE	21 (31.8)	14 (23.7)	0.30	10 (66.7)	25 (22.7)	**<0.001**
Genetic testing						
DSP	54 (81.8)	43 (72.9)	0.23	7 (46.7)	90 (81.8)	**0.002**
Non-DSP	7 (10.6)	15 (25.4)	**0.026**	7 (46.7)	15 (13.6)	**0.001**
PKP2	4 (6.1)	6 (10.2)	0.41	1 (6.7)	9 (8.2)	0.79
DSG2	2 (3.0)	6 (10.2)	0.11	4 (26.7)	4 (3.6)	**<0.001**
JUP	0	3 (5.1)	0.18	2 (13.3)	1 (0.9)	**0.004**
DSC2	1 (1.5)	0	0.56	0	1 (0.9)	0.71
ECG						
**Normal ECG**	4 (6.1)	11 (18.6)	**0.033**	0	15 (13.6)	0.15
**QRS (msec)**	96±15	96±15	> 0.99	98±14	96±15	0.89
**First degree AV block**	6 (9.1)	4 (6.8)	0.60	0	10 (9.1)	0.28
**NSICD**	2 (3.0)	0	0.31	0	2 (1.8)	0.41
**RBBB**	0	4 (6.8)	0.27	1 (6.7)	3 (2.7)	0.19
**LAFB**	7 (10.6)	8 (13.6)	0.41	4 (26.7)	11 (10.0)	0.063
**LPFB**	10 (15.2)	7 (11.9)	0.55	4 (26.7)	13 (11.8)	0.12
**LBBB**	1 (1.5)	0	0.56	0	1 (0.9)	0.71
**Pathological Q waves**	16 (24.2)	16 (27.1)	0.71	7 (46.7)	25 (22.7)	**0.046**
Lateral distribution	7 (10.6)	5 (8.5)	0.47	3 (20.0)	9 (8.2)	0.14
Inferior distribution	7 (10.6)	8 (13.6)	0.52	3 (20.0)	12 (10.9)	0.52

Precordial distribution	1 (1.5)	1 (1.7)	0.87	0	2 (1.8)	0.41
More 2 localizations	1 (1.5)	2 (3.4)	0.17	1 (6.7)	2 (1.8)	0.26
**Fragmented QRS**	28 (42.4)	18 (30.5)	0.42	4 (26.7)	42 (38.2)	0.41
Lateral distribution	3 (4.5)	3 (5.1)	0.73	1 (6.7)	5 (4.5)	0.55
Inferior distribution	18 (27.3)	10 (16.9)	0.18	1 (6.7)	27 (24.5)	0.14
Precordial distribution	1 (1.5)	1 (1.7)	0.87	0	2 (1.8)	0.41
More 2 localizations	6 (9.1)	4 (6.8)	0.60	2 (13.3)	8 (7.3)	0.42
**Global LQRSV**	8 (12.1)	4 (6.8)	0.23	3 (20.0)	9 (8.2)	
**LQRSV in limb leads**	9 (13.6)	9 (15.3)	0.84	3 (20.0)	15 (13.6)	0.54
**Local LQRSV**						
Lateral distribution	12 (18.2)	17 (28.8)	0.15	2 (13.3)	27 (24.5)	0.34
Inferior distribution	11 (16.7)	8 (13.6)	0.56	0	19 (17.3)	0.11
Inferolateral distribution	4 (6.1)	1 (1.7)	0.15	1 (6.7)	4 (3.6)	0.35
Precordial and local	6 (9.1)	6 (10.2)	0.81	2 (13.3)	10 (9.1)	0.37
**QTc (msec)**	408±25	411±24	0.99	412±29	409±24	0.66
**QTc ≥440 msec**	4 (6.1)	6 (10.2)	0.30	2 (13.3)	8 (7.3)	0.42
**Tzou criteria** *	9 (13.6)	10 (16.9)	0.56	4 (26.7)	15 (13.6)	0.19
**R >3 mm V1**	5 (7.6)	5 (8.5)	0.80	3 (20.0)	7 (6.4)	0.051
**R/S ratio ≥0.5 in V1**	21 (31.8)	12 (20.3)	0.13	7 (46.7)	26 (23.6)	**0.048**
**R/S ratio ≥1 in V1**	11 (16.7)	4 (6.8)	0.090	4 (26.7)	11 (10.0)	0.058
**Bayés de Luna criteria** †	3 (4.5)	3 (5.1)	0.73	2 (13.3)	5 (4.5)	0.14
**TWI**	35 (53.0)	23 (39.0)	0.12	11 (73.3)	47 (42.7)	**0.029**
Inferolateral TWI	4 (6.1)	5 (8.5)	0.58	3 (20.0)	6 (5.5)	**0.031**
Anterior TWI	9 (13.6)	2 (3.4)	**0.045**	0	11 (10.0)	0.25
Inferior TWI	1 (1.5)	4 (6.8)	0.082	0	5 (4.5)	0.17
Lateral TWI	7 (10.6)	4 (6.8)	0.32	3 (20.0)	8 (7.3)	0.092
Anterolateral TWI	10 (15.2)	5 (8.5)	0.12	3 (20.0)	12 (10.9)	0.32
Inferior-anterior-lateral TWI	4 (6.1)	3 (5.1)	0.76	2 (13.3)	5 (4.5)	0.14
**NEW ECG CRITERIA**						
**SV1+RV6 ≤12 and RI+RII ≤8 (mm)**	32 (48.5)	23 (39.0)	0.29	10 (66.7)	45 (40.9)	0.060

Data are expressed as n (%) or mean ± standard deviation as appropriate.

AC indicates arrhythmogenic cardiomyopathy; AV, atrio-ventricular; DCM, dilated cardiomyopathy; DSC2, desmocollin-2; DSG2, desmoglein-2; DSP, desmoplakin; JUP, plakoglobin; LAFB, Left anterior fascicular block; LBBB, Left bundle branch block; LPFB, Left posterior fascicular block; LQRSV, low QRS voltages; LV, left ventricular; MAE, Major Arrhythmic Events; NSICD, Non-specific intraventricular conduction delay; NSVT, non sustained ventricular tachycardia; PKP2, plakophilin 2; RBBB, Right bundle branch block; TWI, T wave inversion. * V1R ≥ 0.15 mV and V6S ≥ 0.15 mV; †R/S ratio in V1 ≥ 0.5 and R amplitude in V1 >3 mm

Supplemental Table 4. Clinical, structural and electrocardiographic characteristics of the study population according to the Proband Status.

	**Probands** (n=89)	**Relatives** (n=36)	**P Value**
Age at diagnosis, years	38±15	34±15	0.18
Male	47 (52.8)	17 (47.2)	0.57
Asymptomatic	21 (23.6)	21 (58.3)	**<0.001**
Unexplained syncope	13 (14.6)	2 (5.6)	0.16
NSVT	44 (49.4)	11 (30.6)	0.056
MAE	31 (34.8)	4 (11.1)	**0.008**
ECG			
**Normal ECG**	7 (7.9)	8 (22.2)	**0.026**
**QRS (ms)**	97±15	96±14	0.73
**First degree AV block**	8 (9.0)	2 (5.6)	0.53
**NSICD**	2 (2.2)	0	0.37
**RBBB**	3 (3.4)	1 (2.8)	0.86
**LAFB**	14 (15.7)	1 (2.8)	**0.045**
**LPFB**	13 (14.6)	4 (11.1)	0.61
**LBBB**	0	1 (2.8)	0.11
**Pathological Q waves**	25 (28.1)	7 (19.4)	0.31
Lateral distribution	10 (11.2)	2 (5.6)	0.34
Inferior distribution	11 (12.4)	4 (11.1)	0.84
Precordial distribution	1 (1.1)	1 (2.8)	0.49
More 2 localizations	3 (3.4)	0	0.26
**Fragmented QRS**	36 (40.4)	10 (27.8)	0.19
Lateral distribution	6 (6.7)	0	0.11
Inferior distribution	22 (24.7)	6 (16.7)	0.33
Precordial distribution	2 (2.2)	0	0.37
More 2 localizations	6 (6.7)	4 (11.1)	0.41
**Global LQRSV**	12 (13.5)	0	**0.021**
**LQRSV in limb leads**	16 (18.0)	2 (5.6)	0.075
**Local LQRSV**			
Lateral distribution	20 (22.5)	9 (25.0)	0.77
Inferior distribution	10 (11.2)	9 (25.0)	0.052
Inferolateral distribution	3 (3.4)	2 (5.6)	0.57
Precordial and local	8 (9.0)	4 (11.1)	0.72
**QTc (msec)**	409±26	411±21	0.68
**QTc ≥440 msec**	8 (9.0)	2 (5.6)	0.53
**Tzou criteria***	11 (12.4)	8 (22.2)	0.17

**R >3 mm V1**	5 (5.6)	5 (13.9)	0.12
**R/S ratio ≥0.5 in V1**	28 (31.5)	5 (13.9)	**0.044**
**R/S ratio ≥1 in V1**	12 (13.5)	3 (8.3)	0.42
**Bayés de Luna criteria †**	5 (5.6)	2 (5.6)	> 0.99
**TWI**	48 (53.9)	10 (27.8)	**0.008**
Inferolateral TWI	8 (9.0)	1 (2.8)	0.23
Anterior TWI	9 (10.1)	2 (5.6)	0.42
Inferior TWI	2 (2.2)	3 (8.3)	0.11
Lateral TWI	9 (10.1)	2 (5.6)	0.42
Anterolateral TWI	15 (16.9)	0	**0.009**
Inferior-anterior-lateral TWI	5 (5.6)	2 (5.6)	> 0.99
**NEW ECG CRITERIA**			
SV1+RV6 ≤12 and RI + RII ≤8 (mm)	44 (49.4)	11 (30.6)	0.056
Cardiac magnetic resonance			
LVEDVi (ml/m2)	94.8±24.4	89.7±18.5	0.26
LVEF, %	49.2±10.3	54.5±8.6	**0.007**
RVEDVi (ml/m2)	84.3±20.0	84.3±22.4	> 0.99
RVEF, %	52.5±10.5	54.3±8.7	0.37
Segments with LGE	7±4; 6 (4-10)	5±4; 4 (3-7)	**0.013**
LGE pattern			
- Ringlike	47 (52.8)	19 (52.8)	> 0.99
LGE distribution			
- Subepicardial	67 (75.3)	28 (77.8)	0.77
- Midmural	9 (10.1)	6 (16.7)	0.31
- Transmural	13 (14.6)	2 (5.6)	0.16

Data are expressed as n (%) or mean ± standard deviation or median (Q1-Q3) as appropriate.

AV indicates atrio-ventricular; LAFB, Left anterior fascicular block; LBBB, Left bundle branch block; LGE, late gadolinium enhancement; LPFB, Left posterior fascicular block; LQRSV, low QRS voltages; LVEDVi, left ventricular end-diastolic volume indexed; LVEF, left ventricular ejection fraction; MAE, Major Arrhythmic Events; NSICD, Non-specific intraventricular conduction delay; NSVT, non sustained ventricular tachycardia; RBBB, Right bundle branch block; RVEDVi, right ventricular end-diastolic volume indexed; RVEF, right ventricular ejection fraction; TWI, T wave inversion. * V1R ≥ 0.15 mV and V6S ≥ 0.15 mV; † R/S ratio in V1 ≥ 0.5 and R amplitude in V1 >3 mm

Supplemental Table 5. Univariable and multivariable Cox regression for MAE with ECG parameters, syncope, cardiac magnetic resonance and genetic variables in a primary prevention scenario.

Variable	Univariable HR (CI)	P	Multivariable HR (CI)	P
**ECG variables**				
**LPFB**	3.05 (1.07-8.68)	0.036		
**R/S ratio ≥0.5 in V1**	2.53 (0.96-6.66)	0.06		
**Anterior TWI**	1.67 (0.62-4.53)	0.31		
**SV1+RV6 ≤12 and RI+RII ≤8 (mm)**	1.81 (0.69-4.78)	0.23		
**Clinical, structural and genetic variables**				
**Syncope**	8.42 (3.19-22.22)	<0.001		
**Transmural LGE**	4.77 (1.65-13.77)	0.004		
**LVEF, %**	0.96 (0.92-0.99)	0.049		
**RVEF, %**	0.97 (0.93-1.00)	0.12		
**Non-DSP**	2.40 (0.81-7.11)	0.11		
**Best multivariate model (Harrells' C=0.79, 95% CI 0.66-0.89)**				
**LPFB**			3.98 (1.3-12.0)	0.014
**Syncope**			19.13 (5.8-63.0)	<0.001
**Transmural LGE**			10.57 (2.9-38.0)	<0.001

CI indicates confidence interval; DSP, desmoplakin; LGE, late gadolinium enhancement; LPFB, left posterior fascicular block; LVEF, left ventricular ejection fraction; MAE, major arrhythmic events; HR, Hazard ratio; RVEF, right ventricular ejection fraction; TWI, T wave inversion.

Figure S1. LGE distribution according to the genotype group. Distribution of LGE according to the 17-segment American Heart Association model in DSP and non-DSP genotypes. DSP = desmoplakin; LGE = late gadolinium enhancement**.**

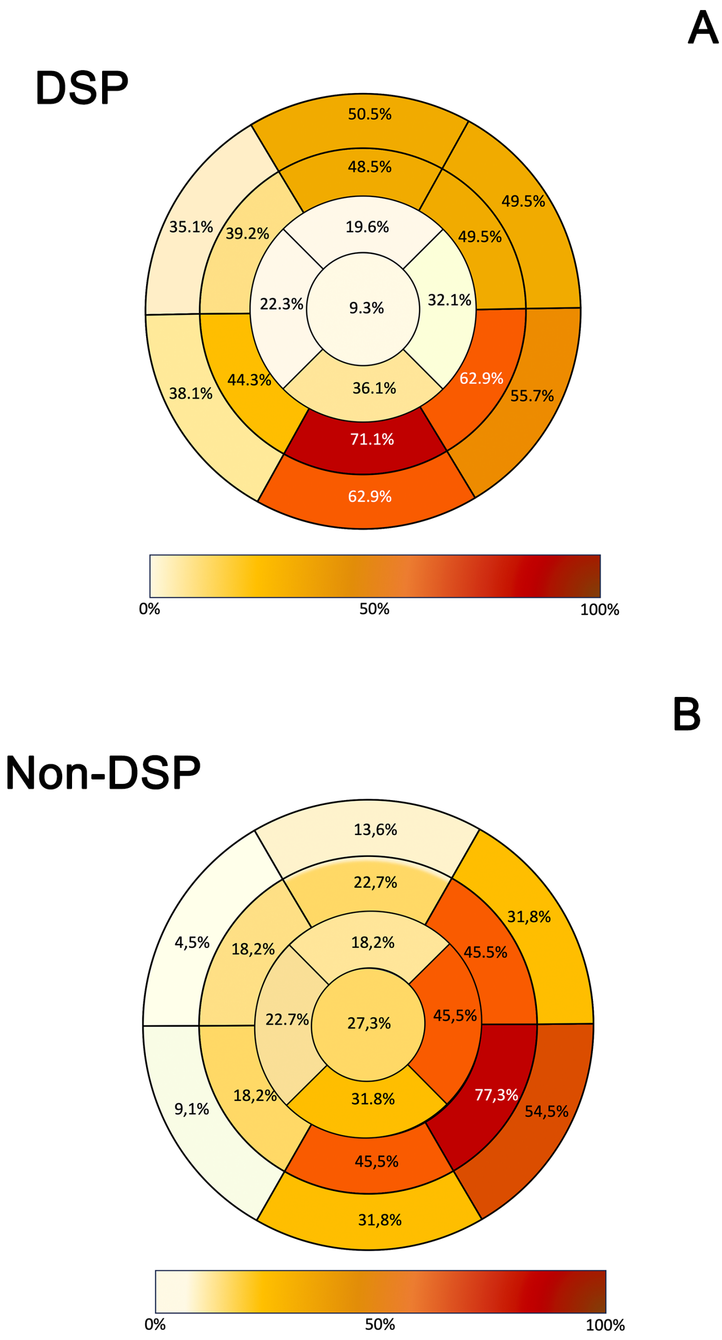

Figure S2. Kaplan-Meier analysis of freedom from the primary endpoint in patients with LPFB versus those without LPFB. *P* obtained with the log-rank test. LPFB= left posterior fascicular block.

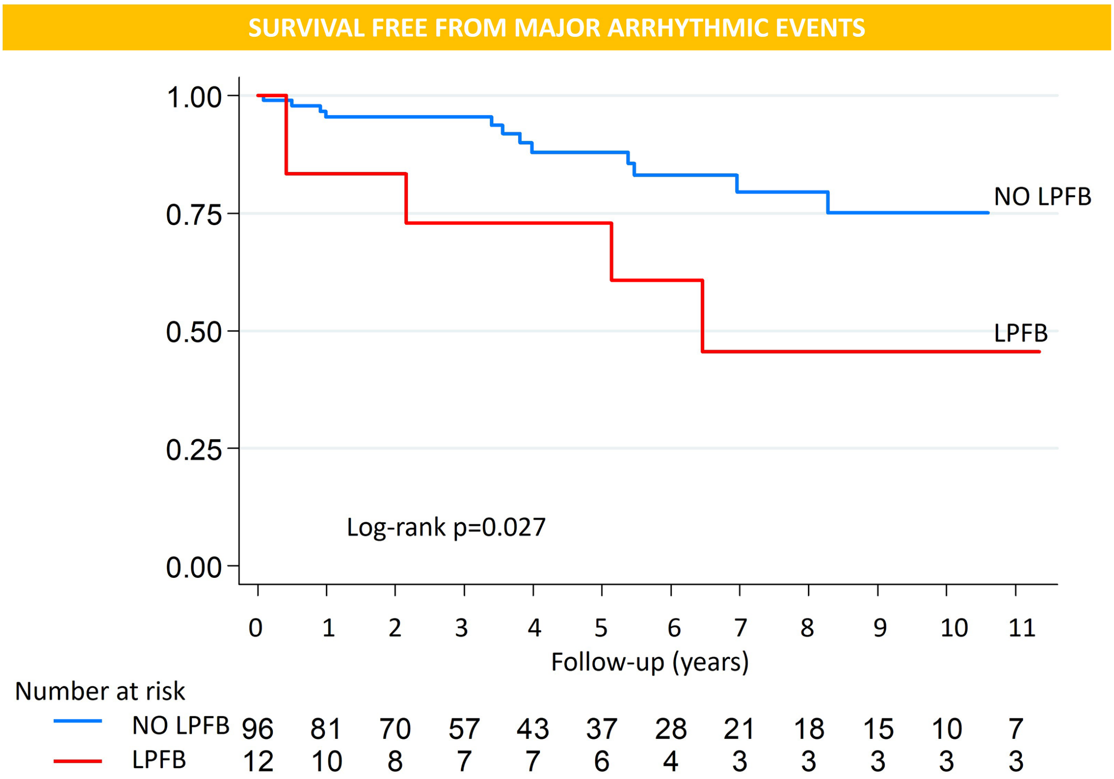

Supplement: Supplementary data [file mmc1.pdf]
